# Respiratory Symptoms in Post-infancy Children. A Dutch Pediatric Cohort Study

**DOI:** 10.3389/fped.2020.583630

**Published:** 2020-12-17

**Authors:** Esther de Vries, Roeland W. N. M. van Hout

**Affiliations:** ^1^Department Tranzo, Tilburg School of Social and Behavioral Sciences, Tilburg University, Tilburg, Netherlands; ^2^Laboratory for Medical Microbiology and Immunology, Elisabeth-Tweesteden Hospital, Tilburg, Netherlands; ^3^Jeroen Bosch Academy Research, Jeroen Bosch Hospital, ‘s-Hertogenbosch, Netherlands; ^4^Centre for Language Studies, Radboud University, Nijmegen, Netherlands

**Keywords:** child, respiratory symptom, season, age, cohort, general population

## Abstract

**Aim:** To study the pattern of respiratory symptoms in children in the general population.

**Method:** We followed a cohort of children for up to 2 years through parents completing weekly online questionnaires in the Child-Is-Ill study (“Kind-en-Ziekmeting” in Dutch); the study was running 2012–2015. Inclusion criteria were “an ordinary child” (according to the parents) and <18 years old at inclusion. We especially encouraged participation of post-infancy children. Age at inclusion, sex, smoking exposure, allergy in the family, and frequent infections in the family were noted. Pearson's correlation, principal component analysis, latent class analysis, latent profile analysis, linear regression, and linear mixed effects regression were used in the statistical analyses.

**Results:** Data were collected on 55,524 childweeks in 755 children (50% girls; median age, 7 years; interquartile range, 4–11 years, 97% ≥2 years at inclusion), with reported symptom(s) in 8,425 childweeks (15%), leading to school absenteeism in 25%, doctor's visits in 12%, and parental sick leave in 8%; symptoms lasting ≥3 weeks were rare (2% of episodes). Linear mixed effects regression showed significant, but only limited, effects of season on the proportion of “symptom(s) reported” per individual child. Only runny nose showed a significant, but very small, age effect. However, the variability between the children was considerable. There were no obvious subgroups of children with specific symptom combinations.

**Conclusion:** In any randomly chosen week, the vast majority of children (85%) in our—mainly—post-infancy cohort derived from the general population did not have any symptom, even in the younger age group, even in winter. The children showed considerable variability; no clear subgroups of symptom patterns could be identified, underlining the difficult position of healthcare providers. These results support our opinion that post-infancy children in the general population should not be evaluated as if they are infants when they have recurrent respiratory symptoms. If they clearly deviate from the above-described most common pattern, it is wise to keep an eye on potential, maybe even rare, serious underlying causes.

## Introduction

Especially young children often suffer from respiratory symptoms; this is a source of worry for their parents and a reason for them to consult a doctor. General practitioners as well as pediatricians see a lot of such children with respiratory symptoms and worried parents in day-to-day practice in the first lines of care. Sometimes, the worried parents have a point: the situation can be more serious (e.g., pneumonia). Guidelines with alarm symptoms have been developed for timely recognition of acute severe disease such as pneumonia; these are helpful and widely used (1). However, most respiratory infections in children are mild and self-limiting; their parents can be reassured. It is not always easy to reassure the parents; the child's symptoms may recur more often, or may last longer, than the parents consider “normal.” In many of these cases, still no in-depth analysis is warranted. Day care attendance, cigarette smoke exposure, asthma, or an allergic predisposition may play a role (2). This can be explained, and measures can be taken.

Nevertheless, frequent recurrence of “everyday” respiratory symptoms can be an indication for serious underlying disease such as antibody deficiency (3). Here, the warning signs are more subtle, hidden in the combination of everyday symptoms presenting in an unusual pattern that experts are trained to recognize, but general physicians may easily miss (4). This may lead to underdiagnosis of treatable disease and development—in the long run—of serious complications such as bronchiectasis (5). However, when should suspicion of serious underlying disease arise? In other words: *what is the normal pattern of respiratory symptoms in children?* This question should be answered bearing the age-related development from infancy to adulthood in mind.

Data that focus on the day-to-day situation in the first lines of care are mainly available for young children, however. Data on post-infancy children are limited. Previous studies on this subject (6–30) mostly included young (≤7 years) (6, 11, 12, 14, 18) or very young (≤3 years) children (7, 9, 10, 15–17, 19, 22–26, 28); besides, half of them were published >10 years ago (Supplementary Table 1). Some of these studies (5/25) only used doctor's files or national health registries (7, 15, 20, 21, 29). For general care, the perspective of parents is actually the most important one: this determines which children are being seen in the consulting room. In addition, in some studies (8/25), the recording of symptoms was performed infrequently: only related to health visits, or a monthly, or even one single questionnaire regarding the past years (6, 7, 10–12, 14, 15, 29). In these studies, symptoms not leading to health visits as well as recall bias may limit the applicability of the results in everyday care.

Therefore, we collected a large cohort of children (*n* = 755) with the aim to study the pattern of respiratory symptoms in children in the general population. We followed them for up to 2 years with weekly parental online questionnaires regarding respiratory symptoms in the past week in their child(ren); 733 (97%) and 590 (78%) of these children were ≥2 and ≥4 years of age at inclusion, i.e., post-infancy, respectively.

## Methods

### Study Design

Inclusion criteria for this online 2-year cohort study were “an ordinary child” according to the parents (meaning “without any major medical problems”; no additional criteria were specified), aged <18 years at inclusion, and informed consent (given through the study website by the parents and/or the child, depending on the child's age). The cohort was not randomly selected. We aimed to obtain as large a cohort as possible and especially encouraged participation of children who were at least 2 years of age at inclusion. Children with the same parents could be included in the study. We included children from February 2012 to June 2013. After a short introductory questionnaire, participating parents weekly received automated email alerts with a link to the question “did your child have any symptoms(s) during the past week?” for 2 years. If the child had symptoms in the past week, additional questions about the type of symptoms (that could be related to respiratory tract infections), doctor visits, child absenteeism, parental leave, and antibiotic use followed (questionnaire in Supplementary Table 2; we deliberately limited the number of questions as much as possible to encourage prolonged participation). All symptoms could be reported as “yes, this symptom was present last week” or “no, this symptom was not present last week,” except fever, which could be reported as “temperature was not taken” as well. The study was advertised through local newspapers (small advertisements), social media (Facebook page), and the internet (Google AdWords); participants were encouraged to spread the word to others (family, friends, work, school). The answers were automatically collected and stored in a coded manner in electronic clinical record forms using the Data Management module of the Research Manager system (Cloud 9 software, Deventer, the Netherlands). Reminder emails were sent manually, if needed. The study was ethically approved by the METOPP (now METC Brabant; nr M362). The protocol was also used in a separate cohort of Down syndrome children (with some additional overall questions) and, as such, was published in BMC Pediatrics (31). The parents and children did not receive any incentive for their participation.

### Statistical Analysis

Data analysis was performed using SPSS25 for Mac and—if specifically mentioned—R version 3.4.4. We analyzed the overall frequency (count), proportion “yes, symptom present” (relative frequency, continuous), and combination(s) of (categorical) symptoms (upper respiratory tract: cough, runny nose, blocked nose/general: headache, fever/lower respiratory tract: dyspnea/ENT: throat ache, hoarse voice, earache, ear discharge; present/not present; binary), and the effects thereon of age (three age groups), sex (boy/girl; binary), season (spring/summer/autumn/winter; categorical), smoking exposure (in the house by family/in the house by visitors/only outside/no; categorical), and family history of allergy or of frequent infections (yes/no; binary), as well as their relation to doctor visits (yes/no; binary), antibiotic use (yes/no; binary), school absenteeism (yes/no; binary), and parental sick leave (yes/no; binary).

Parents of children with more symptoms might be inclined to stop (the burden of) study participation sooner, but the opposite was the case in a study on asthmatic children (32). We evaluated the effect of missing data and being siblings using Pearson's correlation (*p* < 0.05 significant; 0.3 < *r* < 0.5 effect size moderate).

We analyzed the data in two steps. As first step, we handled each childweek as a separate data-collection entry. We first summarized the data of all children over all reported childweek(s). Next, we summarized the data of all children per calendar week, aggregating their data of the same calendar weeks from 2012 to 2015 (i.e., each year is subdivided into 52 calendar weeks, calendar week 1 containing ≥4 days of each new year). These outcomes are primarily descriptive (33) and included means, medians, and percentages, and extensive visualization of the data. This enabled us to present plain overviews and track data patterns.

As second step, we took the longitudinal character of the data per individual child into account. We counted the duration of symptom blocks per symptom type (1 week, 2 consecutive weeks, etc.; for this calculation, missing childweeks during participation were interpreted as “no symptoms present” because this was by far the most common value). We calculated the proportion of childweeks with symptoms per total reported childweeks per individual child per symptom type (continuous data, range between 0 and 1) over all reported weeks (year round) as well as per separate season. Next, we divided these results into three age categories (<5, 5–10, ≥10 years at inclusion), a division roughly corresponding to “preschool,” “school,” and “teenage” years.

To identify subgroups of children focused on their symptom patterns, we performed latent profile analysis (LPA) for continuous data using the mclust package in R for model-based clustering, classification, and density estimation based on finite Gaussian mixture modeling (34) and latent class analysis (LCA) for binary data with the poLCA package in R using the Bayesian information criterion (BIC) to select the best model (35). Dimensionality reduction regarding symptom patterns was performed with principal component analysis (PCA) using a correlation matrix with extraction based on eigenvalues >1 and a maximum of 25 iterations for convergence, with the varimax with Kaiser normalization as rotation method.

We applied linear mixed effects (LME) regression and linear regression (LR) to distinguish data patterns and to investigate explanatory factors. LME was performed using the package lme4 in R with child as random factor (36). LME can handle missing data because it uses maximum likelihood estimation. We used the logit values of the proportions p [=ln(p/(1 – p)] of symptoms as the dependent variable, adding 0.005 to 0 values and subtracting 0.005 from 1 values. We primarily used the Akaike information criterion (AIC) to evaluate the regression models. Next to season (spring, summer, autumn, winter), we investigated five other potential predictors (the independent variables) in the regression analyses: age category at inclusion (<5, 5–10, and ≥10 years), sex, smoking exposure, allergy in the family, and frequent infections in the family. We analyzed for main effects as well as for potential interactions between these predictors. For additional overall analyses on the proportion of childweeks with symptom(s) over the whole data collection, we used LR with the lm function in R, using logit values of the proportions of symptoms in the same way as in the LME analysis. We used the R package lsmeans to perform pairwise comparisons, applying the Tukey method, to establish which differences between means were significant.

## Results

### Composition of the Cohort

**Figure 1:**
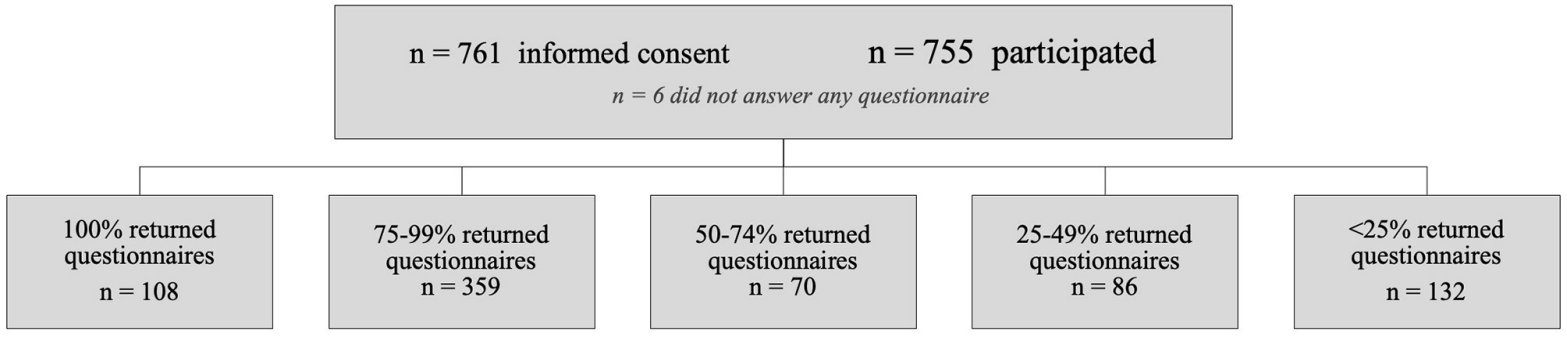
Overview of the reported childweeks obtained in the cohort. Questionnaires on 55,524 childweeks were obtained out of the potential 78,520 (70.7%). Parents sometimes missed reporting one or more childweeks (*n* = 6,773 weeks); in addition, some parents entirely stopped participation before the 2 years had been completed (*n* = 16,223 weeks missing).

Parents gave informed consent for 761 children from 497 families (381 girls, 50.1%; median age at inclusion, 7 years; interquartile range, 4–11 years; 97% ≥2 years at inclusion; median, 1; mean, 1.6; range, 1–4 participating children per family). For six children, the parents never answered any questionnaire. In the remaining 755 children, parents reported atopy in the family in 470 children (62.3%; 1.1% unknown) and frequent infections in the family in 40 children (5.3%; 2.4% unknown). Of the households, 85.7% were reported to be completely smoke free [~25% of Dutch adults smoke^1^]. In addition, in 95.5%, parents reported their ethnicity as “original Dutch descent” [~77% of the Dutch population is of original Dutch descent^2^]. Finally, the participants were scattered throughout the country, but 37.5% resided in the greater ‘s-Hertogenbosch area. Data were obtained on 55,524 out of the potential 78,520 (=2 × 52 × 755) childweeks (70.7%; Figure 1).

The correlation between the proportion of missing questionnaires and the proportion of childweeks with “any symptom” (per child) in our study was moderate (Pearson's *r* = −0.42, two-tailed, *p* = 0.01); there was no significant correlation for sex, age at the start of the study, ethnicity, smoking exposure, infection in the family, allergy in the family, and residence. Therefore, we did not take dropout into account as confounding variable in our statistical analyses.

### Step 1: Childweeks With Symptoms

**Figure 2:**
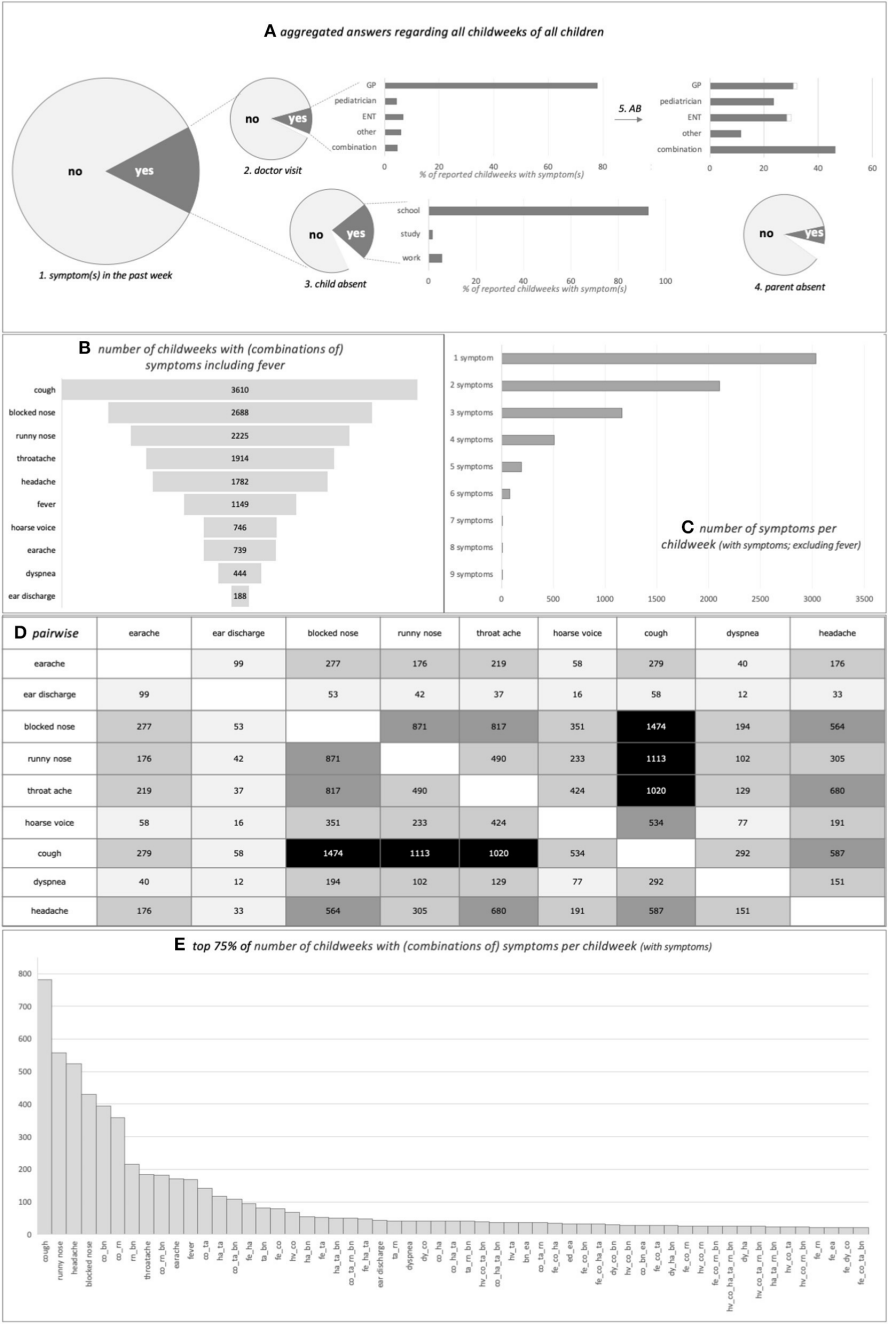
The various parent-reported (combinations of) symptoms in the cohort in all childweeks taken together. **(A)** From left to right: 1, “any symptom(s) present in the past week (yes/no)” is shown. When the answer was “yes:” 2, “did the child visit a doctor” and if so “was this a GP/ENT/other/combination (percentages in bar chart);” 3, “was the child absent from school/study/work (percentages in bar chart);” and 4, “did the parent have to stay home from work” is shown. The top right bar chart shows the percentage of children who received an antibiotic prescription (5). The size of the pie charts is proportional to the relevant number of childweeks per item. Symptoms “yes,” *n* = 8,425 childweeks (15.2%), leading to child's absenteeism in 25.4%, parental sick leave in 8.1%, doctor visit in 11.8%, and antibiotic prescription in 26.3%. White area in graphs = unknown (*n* = 587 childweeks child's absenteeism unknown, *n* = 558 childweeks parental sick leave unknown, *n* = 118 childweeks doctor visit unknown, *n* = 242 childweeks antibiotics prescribed unknown; antibiotics prescribed, but doctor unknown is not shown in antibiotics graph, *n* = 4 childweeks). AB, antibiotics; combination, more than one doctor was visited; ENT, ear–nose–throat specialist; GP, general practitioner; other, another doctor than GP/pediatrician/ENT was visited. **(B)** Number of childweeks where the symptom was reported (including fever). **(C)** Number of childweeks where one to nine symptoms were reported in the same week (excluding fever). **(D)** Number of childweeks where the symptom on the one axis was reported concurrently with the symptom on the other axis (either simultaneously with or not simultaneously with other symptoms); a larger number is reflected in darker shading of the cell (excluding fever). **(E)** Top 75% of number of childweeks with (combinations of) symptoms per childweek with symptoms (including fever). X-axis combination(s) of symptom(s): bn, blocked nose; co, cough; dy, dyspnea; ea, earache; ed, ear discharge; fe, fever; ha, headache; hv, hoarse voice; ta, throat ache; rn, runny nose.

Figure 2A shows the frequency of all childweeks taken together with parentally reported symptom(s) and related actions. The majority (84.8%) of childweeks was without any symptom. Having symptom(s) led to the child's absence from school, study activities, or work in 25.4% of childweeks (7.0% unknown). Parents consulted a doctor for these symptom(s) only 11.8% of the time (1.4% unknown); this was often a general practitioner (GP). Parents stayed home from work because of the child's symptoms 8.1% of the time (6.6% unknown). Once a doctor was consulted, antibiotics were prescribed in 26.3% (0.7% unknown). Parents reported “temperature was not taken” in 38.1% of childweeks with symptoms in the past week (4.1% unknown); therefore, we did not include fever in the analyses in Step 2 below. The observed pattern suggests that parents are more inclined to take their child's temperature in case of dyspnea and less in case of runny nose, blocked nose, or cough (data not shown).

The various symptoms did not occur with the same frequency. The children had only one symptom in 42.7% of the reported childweeks with symptom(s). Some symptoms, and combinations of symptoms, were much more frequent than others (Figures 2B–E). Having only a cough, or a runny nose, or a headache, or a blocked nose comprised one-third of the childweeks with symptoms; one-half additionally comprised combinations of cough, runny nose, and blocked nose and having only a throat ache, or an earache, or a fever.

Next, we grouped the data together per calendar week (1–52, see *Methods*) to obtain an overall insight in the roles of age, sex, and season. The pattern of having symptom(s) in the past week suggested some seasonal influence, with symptom frequency being low year round but declining from winter to autumn to spring to summer, albeit for some symptoms (e.g., cough) more so than for others (e.g., ear discharge); this seasonal influence seemed most pronounced in younger children (Supplementary Figure 1). The pattern of reported symptoms between boys and girls did not suggest any clinically relevant difference (data not shown).

### Step 2: Analyzing Patterns and Investigating Explanatory Variables

Longitudinal analysis of the data gives insight into the duration of (combinations of) symptoms per each individual child and enables classifying subgroups of children regarding the frequency, type, and duration of (combinations of) symptoms, and analysis of symptom predictors.

#### Incidence and Duration of Symptoms

**Figure 3:**
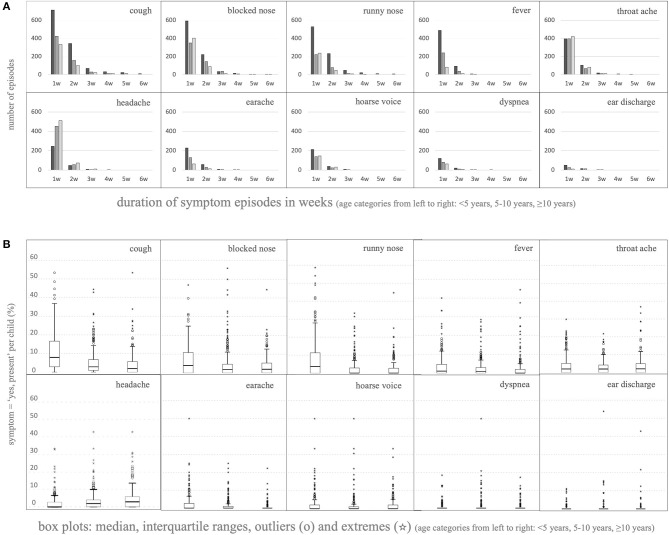
Longitudinal analysis of the various parent-reported (combinations of) symptoms per individual child. **(A)** Duration of episodes of consecutive childweeks counted per individual child depicted up to six consecutive weeks; depicted per symptom, either occurring singly or together with other symptom(s) (for all data see Supplementary Table 3). For this analysis, episodes preceded and/or followed by a missing value (week not reported, or before the start or after the end of the study) were included in the count as if the missing value was “no symptoms in that week.” Thus, it could be that, in reality, the curves of episode duration are shifted slightly toward longer episodes. **(B)** Boxplots showing the distribution of the percentage of “yes, symptom present” per total reported childweeks counted per individual child per symptom [box = p25→p75, whiskers at 1.5*(p75–p25), outliers and extremes outside the whiskers at >1.5 ∨ <3*(p75–p25) (◦); or >3*(p75–p25) (*), respectively].

Counting the duration of symptom blocks per individual child per symptom type showed that most symptom episodes only lasted 1 or 2 weeks, but longer episodes did occur, more often in younger children [Figure 3A, depicted per symptom, either occurring singly or together with other symptom(s); more details in Supplementary Table 3]. Parents reported symptoms in ≤5% of childweeks (equivalent of <2–3 weeks/year) in 32% of children who were <5 years at inclusion. For each symptom type, there was also a large subset of children in each age group that (almost) never suffered the symptom, leading to extremely low medians and interquartile ranges (Figure 3B). Older children (≥5 years at inclusion) showed skewing toward lower symptom frequency, in concordance with the analysis of the separate childweek data in *Composition of the Cohort*. However, they also showed subgroups with higher frequency: parents reported symptoms in >15% of childweeks (equivalent of >8 weeks/year) in 16% of children who were ≥5 years at inclusion. Thus, the variability between the children was considerable.

#### Subgroup Analysis

Using LPA and PCA over all individual children on the symptom variables showed that no subgroups of children with clearly distinguishable symptom patterns could be discerned in the data (Supplementary Table 4). LPA resulted in two to nine best fitting numbers of clusters depending on the combination of age category and season; however, plotting the classifications showed that there was no clear pattern discernible in the symptom combinations in these clusters. Clusters overlapped, and many cases did not fit into the cluster ellipses. LPA using only the symptoms in the relevant components identified in the PCA showed that there was no clear pattern in any of those symptom combinations as well (data not shown).

We selected the children who were outlier and/or extreme in ≥1 symptom type in Figure 3B for separate analysis. LCA showed a two-cluster solution yielded the best fit for classification (BIC, 3,846.289; estimated class population shares 0.38 and 0.62 for class 1 and 2, respectively). PCA resulted in two components located to the left of where the scree plot tapered off: “child is an outlier/extreme for cough and blocked nose and runny nose” (loadings 0.710, 0.763, 0.705), and “child is an outlier/extreme for throat ache and headache” (loadings, 0.613, 0.686). Separate PCAs for children <5 and ≥5 years at inclusion showed a difference, with two components <5 years (*n* = 169 children; “child is an outlier/extreme for cough and blocked nose and runny nose;” loadings 0.742–0.784–0.620; “child is an outlier/extreme for throat ache, headache and earache,” loadings 0.711–0.621–0.645) and one component for ≥5 years (*n* = 226 children; “child is an outlier/extreme for blocked nose and runny nose,” loadings 0.738–0.718).

#### Regression Analysis: Investigating the Explanatory Variables

**Figure 4:**
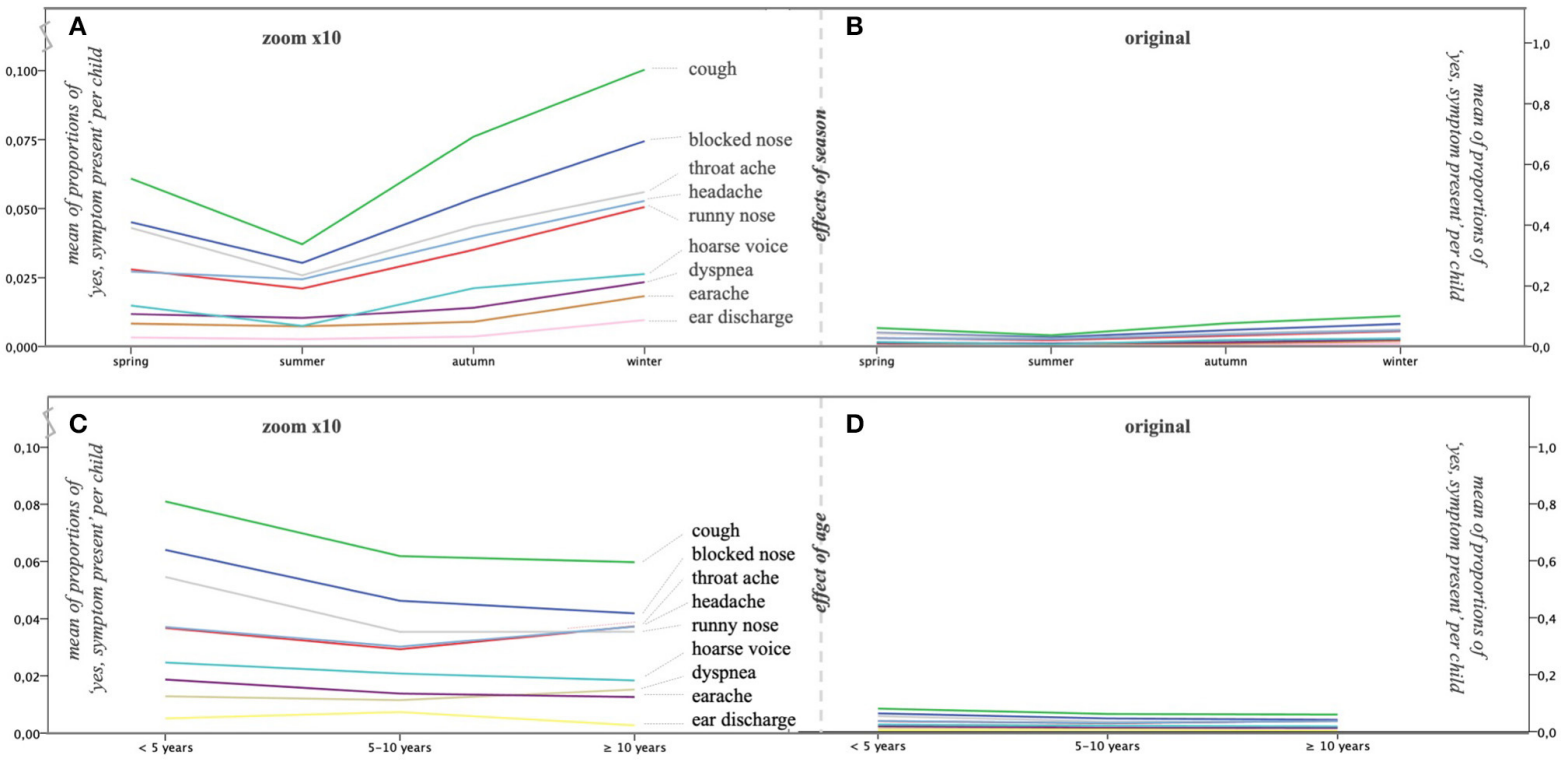
Choosing the right perspective to evaluate the effects of season and age. The mean proportion of childweeks with “yes, symptom present” calculated per individual child per all reported child weeks is shown on the y-axis [**(A,C)**: y-axis on the left; **(B,D)**: y-axis on the right]. Zooming in on the symptoms **(A,C)** shows the effects; zooming out to the original size **(B,D)** shows the effects in the right perspective: they are limited. **(A)** The effects of season (x-axis) are clearly visible when zoomed in (× 10), albeit more pronounced for some symptoms than for others. **(B)** When zoomed out to the original size, it is clear that the effects of season are small. **(C)** When zoomed in (× 10), it is clear that an effect of age (x-axis) is present but only for a few symptoms (see also Table 1). **(D)** When zoomed out to the original size, it is clear that the effect of age is very limited.

We ordered the childweek data per individual child on season, separately computing the logit of the proportion of childweeks with symptoms in each of the four seasons for nine symptom types (excluding fever). The effects of season on the nine symptom types are clearly visible in Figure 4A, albeit more pronounced for some symptoms than for others but small (Figure 4B): even in winter, most childweeks were without symptoms. This is in accordance with the descriptive per-calendar-week analysis in *Step 1: Childweeks With Symptoms*. Figure 4C shows the effect of age, which is very limited (Figure 4D).

We ran LME analyses for the nine symptom types (excluding fever; Table 1A). We first entered season and age and their interaction and selected the model with the lowest AIC. All symptoms had a significant main effect for season, confirming that the differences in Figure 4A are significant. Only runny nose showed a significant main age effect. Sex was never significant. The effects of allergy in the family, frequent infections in the family, and smoking exposure of the child were low to absent (one significant main effect of allergy for cough; see Table 1A).

Table 1Distinguishing data patterns and investigating explanatory factors.

**Table:** 

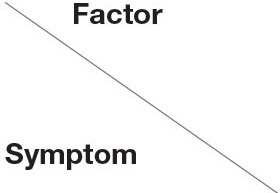	**Main effect season**	**Less symptoms**	**More symptoms**	**Most symptoms**	**Main effect age**	Interaction season*age	**Main effect allergy in the family**	**Main effect frequent infections in the family**	**Main effect smoking in the house**
**A**									
Cough	X	Summer	Spring = autumn	Winter	0	0	X	0	0
Blocked nose	X	Summer	Spring = autumn	Winter	0	0	0^a^	0	0
Runny nose	X	Summer	Spring = autumn	Winter	X	0	0	0	0
Headache	X	Summer	Spring = autumn	Winter	0	0	0	0	0
Throat ache	X	Summer = spring	Autumn	Winter	0	0	0	0	0
Dyspnea	X	Summer = spring	(Autumn)^b^	Winter	0	0	0	0	0
Ear discharge	X	Summer = spring	(Autumn)^b^	Winter	0	0	0^a^	0	0
Earache	X	Summer = spring = autumn		Winter	0	0	0	0	0
Hoarse voice	X	Summer = spring = autumn		Winter	0	0	0	0	0

**Table:** 

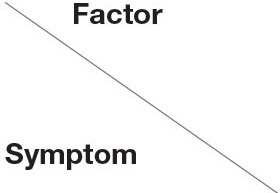	**Main effect age**	**Comparison <5 vs. 5–10 yrs of age**	**Comparison 5–10 vs**. **≥10 yrs of age**	**Comparison <5 vs**. **≥10 yrs of age**	***R***^**2**^ **(in %)**	**Main effect allergy in the family**	**Main effect frequent infection in the family**	**Main effect smoking in the house**
**B**								
Cough	X	0	0	X	1.6	0	0	0
Blocked nose	X	X	X	0	2.5	X	0	0
Runny nose	X	X	X	0	1.3	0	0	0
Headache	0	0	0	0	0.6	X	0	0
Throat ache	0	0	0	0	1.0	X^c^	0	0
Dyspnea	0	0	0	0	–	0	0	0
Ear discharge	0	0	0	0	–	0	0	0
Earache	0	0	0	0	–	0	0	0
Hoarse voice	0	0	0	0	–	0	0	0

***(A)** Mixed linear regression modeling (LME) on the logit of the proportions of childweeks with “yes, symptom present” analyzed per season. X = significant main effect, 0 = no significant main effect (meaning all symptom frequencies are significantly affected by season, but only runny nose by age and cough by allergy in the family when analyzed separately). Summer/spring/autumn/winter = significant differences in seasonal pattern based on pairwise comparisons using the lsmeans R package. The maximal seasonal difference was found for cough, between 4%, (95% CI 3.1–4.3%) in summer vs. 10% (95% CI 8.9–11.3%) in winter. The maximal age difference was found for blocked nose, between 6% (95% CI 5.2.1–7.6%) <5 years vs. 4% (95% CI 3.4–4.9%) ≥10 years*.

a *No interactions were found, except for cough (more frequent in children with allergy in the family), and for blocked nose and ear discharge (more frequent in children without allergy in the family, but only in the winter)*.

b Symptom frequency is in between summer/spring and winter, but the difference is not significant for comparisons with autumn. **(B)** Linear regression analysis (lm in R) per symptom on the logit of the proportions of childweeks with “yes, symptom present” analyzed year-round (all childweeks together per individual child). X = significant effect (meaning those symptoms were significantly more frequent with lower age or with allergy in the family, except for

c *throat ache which was significantly more frequent in children without allergy in the family). R^2^ = the percentage of the explained variance. Age analyzed also in pairwise comparisons between the three age groups using the lsmeans R package. Maximal seasonal difference was found for cough: between 4%, (95% CI 3.1–4.3%) in summer vs. 10% (95% CI 8.9–11.3%) in winter. Maximal age difference was found for blocked nose: between 6% (95% CI 5.2.1–7.6%) <5 years vs. 4% (95% CI 3.4–4.9%) ≥10 years. Yrs, years. P < 0.05 were considered significant*.

We tested for significant differences between season pairs per separate symptom using the package lsmeans in R. Cough, blocked nose, runny nose, and headache have the same pattern: summer has the lowest proportion of symptoms and winter the highest, autumn, and spring being in between (albeit the differences are small, Figure 4B). All other patterns similarly distinguish summer and winter as different seasons, but the position of autumn and spring is more variable (Table 1A).

It is important to note that Figure 4 only shows the *central tendency* in the cohort; the *variability* is shown in Figure 3B. That variability in proportions of symptoms was large and increased by splitting up the data in seasons. Therefore, weaker effects may have been overshadowed by the variability within and between individual children. A way to reduce the impact of within-child variability is to aggregate the data over the whole period of observation (year round) to better be able to scrutinize the fairly weak effects of age, allergy in the family, frequent infections in the family, and smoking exposure. We therefore repeated the regression analysis for each symptom separately, the dependent variable being the logit of the proportion of symptoms over the whole period of observation using standard multiple regression analysis (function lm in R). We first entered age and added the other effects next, including their interaction with age if age was significant. By backward selection, we selected the most parsimonious model (using *R*^2^ change, α = 0.05, as criterion; Table 1B). There was only a significant age effect in three symptoms. There were no interactions with the three other factors. We again applied pairwise comparisons to explore the age effect of the three age groups in more detail. The comparisons indicate a younger age group effect for cough, blocked nose, and runny nose. For three symptoms, we saw an effect of allergy in the family. All effects were weak, as indicated by the low percentages of explained variance (*R*^2^), varying between 0.6 and 2.5% for the five symptoms that had a significant effect.

## Discussion

### Main Messages

Our most important observation is that, in any randomly chosen week, the vast majority of children in our cohort (85%) did not have any symptom, even in the younger age group, even in winter. As a result, and contrary to what is generally expected, the influence of season on symptom frequency was limited, and the influence of age was very small (Figure 4, Table 1). However, the variability between the children was considerable, and this was the case in all age groups (Figure 3B). Although it is attractive to presume that the children with more frequent symptoms were suffering from—maybe undiagnosed—atopic diseases, this is not supported by our data. We did not find an effect of allergy in the family on most symptom frequencies. Most episodes with symptoms lasted only 1–2 weeks (84% of episodes in children <5 years, 92% of episodes in children 5–10 years, and 93% of episodes in children ≥10 years at inclusion); longer episodes were rare but did occur (2% of episodes were ≥3 weeks; Figure 3A; there may be some underestimation here because of our calculation method, see *Methods* and *Results*).

Even if children had symptoms in a childweek, in 88% of cases, they did not visit a doctor, although they stayed home 25% of the time and parents interrupted their daily duties 8% of the time (Figure 2). Many symptom episodes may have been the result of non-infectious causes (migraine, allergy, etc.; see also *Introduction*), and since many respiratory infections are self-limiting and viral in origin (37, 38), it is likely that some children who did visit a doctor during a childweek with symptoms received unnecessary treatment: in 26%, the visit resulted in the prescription of an antibiotic.

Thus, post-infancy children who are seen in the consulting room with symptoms that could be related to respiratory infections already form a highly selected group, even in general care. It is important for all healthcare providers who see a lot of children with respiratory episodes (39) to realize this: if post-infancy children clearly deviate from the above-described most common pattern, it is wise to keep an eye on potential, maybe even rare, serious underlying causes. On the other hand, the huge variability with many outliers we found in this cohort of ordinary children from the general population underscores the difficult position of healthcare providers: it may well be the extreme of normal. Unfortunately, but also remarkably, it was not possible to identify clearly distinguishable subgroups of children in the cohort based on their symptom type pattern as a whole, nor when split into age groups or evaluated per separate season. Only the outliers (Figure 3B) showed two clusters in the LCA and one (≥5 years at inclusion) or two (<5 years at inclusion) components in the PCA, but this seemed to be based mainly on clustering of children who have at least one of these symptoms very frequently. All this does not really help healthcare providers in their evaluation of post-infancy children with respiratory symptoms.

### Comparison With the Literature

Most studies (Supplementary Table 1) have not related the relative proportion of time periods with symptoms to those without symptoms, except one (13). That study did show a decreasing proportion of respiratory episodes with age and a relationship with season in the same low ranges that we observed. This study focused on gastroenteritis related to water supply but included episodes of runny nose, sore throat, or cough with or without fever or chills in the weekly parental diaries. Extrapolating the results from the other studies (all focused on ENT and/or respiratory episodes) to relative proportions shows a similar picture to our study results when including only the post-infancy children: most of them do not have respiratory symptoms most of the time, and the effects of season and age on those relative proportions are limited.

### Strengths and Limitations of the Study

The strengths of our study include a large overall cohort size, an unusually long follow-up period, and weekly data collection, which minimizes recall problems.

Our study also has limitations. First, the cohort was not completely unbiased; household smoking was underrepresented and residing in the greater ‘s-Hertogenbosch area as well as original Dutch descent were overrepresented. In addition, missing childweeks and premature discontinuation of the study may have led to overrepresentation of children with more frequent symptoms and some underestimation of the duration of symptom blocks (for details see *Results*). We chose not to collect in-depth medical history data to keep the burden of participation in this long-term survey study as low as possible. This means that we cannot relate the results of our study to potential influence of differences in medical history in the participating children. We also chose to include all the children in the family who were “ordinary children” according to the parents because we wanted to study the real-life situation as faced in the first lines of care (where patients are not selected on the basis of study criteria), but this may have led to some bias for two reasons. First, siblings share the same family history regarding, e.g., allergy and asthma, and second, they may transmit respiratory viruses more easily among each other and thus influence each other's respiratory symptom frequencies. Obviously, the results should also be assessed in the light of the study background: a high-income country harboring an equally accessible healthcare system for all inhabitants with GPs in the role of gatekeeper.

Finally, it is important to keep in mind that we focused our research on post-infancy children (only 0.7% of childweeks were reported for children <2 years of age at inclusion). This, in fact, is a strength of our study. However, it also means our data cannot and should not be extrapolated to infants.

## Conclusion

We weekly followed a large cohort of Dutch children for respiratory symptoms for up to 2 years; the children were mainly (97%) post-infancy (≥2 years of age) at inclusion. Most childweeks were without symptoms in the majority of these children, also in the younger age group, even in winter. Most symptoms lasted only 1 or 2 weeks. Even when there were symptoms, most of these children did not visit a doctor. Moreover, the effects of season on symptom frequency were limited, and the effect of age was very small. It is important to realize this when seeing a post-infancy child with respiratory symptoms in the consulting room: for healthcare providers, this is “business as usual,” and they are inclined to judge the situation colored by their experience with infants, but for the post-infancy child, this is an exceptional situation that merits attention. However, even though most post-infancy children do not come for a visit when they have respiratory symptoms, one still sees a lot of them in most bustling practices. Additionally, the considerable variability between the children, as well as the lack of clear subgroups based on age and/or symptom(s) found in our study, underline the difficult position of healthcare providers. Nevertheless, our results offer a wider view on the pattern of respiratory symptoms in post-infancy children in the general population. This can be of help to adequately evaluate post-infancy children in the right perspective when they are visiting general care.

## Data Availability Statement

All datasets generated for this study are included in the article/Supplementary Material.

## Ethics Statement

The studies involving human participants were reviewed and approved by METOPP now METC Brabant (PO Box 90151, 5000 LC Tilburg, Netherlands; info@metcbrabant.nl). Written informed consent to participate in this study was provided by the participants' legal guardian/next of kin.

## Author Contributions

EV designed and executed the study and wrote the consecutive versions of the manuscript. EV and RH performed the statistical analyses, drafted the figures and tables, and critically reviewed and commented all versions. Both authors agree to be accountable for the content of the work.

## Conflict of Interest

The authors declare that the research was conducted in the absence of any commercial or financial relationships that could be construed as a potential conflict of interest.

## Footnotes

^1^https://opendata.cbs.nl/statline/#/CBS/nl/dataset/83021NED/table?dl=1BB38 (accessed June 28, 2019).

^2^https://opendata.cbs.nl/statline/#/CBS/nl/dataset/37296ned/table?dl=107C4 (accessed June 28, 2019).
