# Supplementary material for: Respiratory Symptoms in Post-infancy Children. A Dutch Pediatric Cohort Study
Source: Front Pediatr. 2020 Dec 17;8:583630. doi: 10.3389/fped.2020.583630 (PMC7773946; doi:10.3389/fped.2020.583630)
Supplement: Supplementary file 1 [file Table_1.pdf]

Supplementary Table 1. Selected population studies on respiratory symptoms in children

PMID[1]	Ref[2]	Country	Population	Period (yrs)	Period (FU)	Age	Number	Methods	Results
		where study was performed	*where cohort was recruited*	*in which the cohort was recruited*	*of follow-up of the cohort*	*of cohort participants*	*of cohort participants*	*cohort selection; data collection; perspective (parents or professionals)*	*summary of reported respiratory symptoms*
2732519	6	USA	Large city, children <3 ms at 1st visit to two separate pediatric practices (1 urban, 1 private suburban)	1975-1977	1-7 yrs	0-7 yrs	877	Predefined parental questionnaires at each pediatric visit, regardless of its primary purpose	At 1 yr of age, 62% had ≥1 otitis media episode, 17% had ≥3 episodes; by 3 yrs of age, 83% had ≥1 episode, 46% had ≥3 episodes; peak incidence was during 6-12 ms of life (increased risk for males, siblings with recurrent otitis, early occurrence of otitis, not being breast fed)
2729259	7	USA	Large city, pediatric health organisation	1980-1984	1 yr	0-1 yrs	1179	Active recording of symptoms during doctor visits	32.88% doctor visit for resp illness; 60% of lower resp infection was bronchiolitis
9604409	8	Netherlands	Nationwide, 161 GP practices, 100 children per practice, 70% of selected families participated	1987-1988	3 wks	0-14 yrs	1805	Randomly selected; diary, structured questionnaire parents, phone calls; all seasons included (primary study endpoint ‘illness’)	Common colds, flu-like and airway symptoms were most frequent, especially in young children, and in autumn and winter. 60% had symptoms during the 3 weeks of FU, 1 in 6 of these visited their GP
7702884	9	UK	Mixed urban and rural area, birth cohort from two neighbouring general practices	1987-1989	2 yrs	0-2 yrs	333	Diary parents (11,500 childdays of observation)	Prevalence of cold, snuffles or sneezing 9.5/100 child days of observation (increased risk with siblings in the family).

10353923	10	USA	Large city, outpatient clinic for pregnant women	1991-1994	6 ms	0-6 ms	596	Monthly structured questionnaire parents (primary study endpoint otitis media)	Focus on risk factors influencing frequency and early occurrence of otitis media (day care, airway infections, family size & history, and season identified as such)
11031406	11	Norway	Large city, national census registry	1996	n.a.	0-5 yrs	3754	Single structured questionnaire parents; retrospective (‘last month’ and ‘last year’)	Focus on risk factors for airway infections (atopy, low birth weight, day care, family size, age, season identified as such)
29447878	12	Denmark	National birth cohort, 30% of the eligible population	1996-2002	7 yrs	0-7 yrs	54,549 (post-natal cohort)	Telephone interview at 12 & 30 wks of pregnancy, and at 6 & 18ms of age; questionnaire at 7 yrs of age	Focus on otitis media, 40% ≥1 10% >3 episodes at 18ms, 24% >3 episodes at 7 yrs; increased risk multiple episodes with short breastfeeding & early daycare
14705301	13	Australia	Large city	1997-1998	15 ms	all ages (children and adults)	1460	Weekly structured questionnaire parents (primary study endpoint gastroenteritis) (childweeks of observation not separately described)	Focus on gastroenteritis related to water supply. ≥80% reported ≥1 resp episode in 15ms; average 2.2 episodes/person/ yr, duration 6.3 days; more episodes in younger children; doctor visit for 1 in 4 episodes
19159954	14	Germany	Four large cities, birth cohort	1997-2005	6 yrs	0-6 yrs	3097	Monthly questionnaires parents	66.7% ≥1 otitis media episode, 13.5% ≥1 pneumonia episode in 6 yrs, peak in 2nd yr, influence of season and gender; half had antibiotics for otitis in 1st yr
21886448	15	Canada	Large region, health registries	1999-2000	3 ys	0-3 yrs	50474	Linking of health registry data, doctor visit data	48.6% ≥1 doctor visits for otitis media, highest incidence in 1st & 2nd yr; influence by family and social factors

17123315	16	Switzerland	Large city, maternity hospitals & obstetrician's practices, BILD birth cohort	1999-2005	1 yr	0-1 yrs	195	Weekly phone calls (10,275 childweeks of observation)	≥1 resp episode in 46% (severe) and 93% (all) children, in 9.9% of person-weeks; 90% had symptoms in ≤12 wks
30474038	17	Switzerland	Large city, maternity hospitals & obstetrician's practices, BILD birth cohort	1999-2015	6 yrs	0-6 yrs	369	Weekly telephone interview in 1st year of life on respiratory symptoms and infections (16,864 childweeks of observation)	Low, intermediate and high risk phenotype of symptom dynamics in 1st yr of life, high risk phenotype increased risk of wheezing at 6 yrs (OR 3.01)
15790323	18	Australia	Large city	2001	5 ms	12-71 ms	118	Diary parents, phone calls (477,3 childmonths of observation)	0.53 resp episode/chld-month; risk factors age, family size, day care; doctor visit for 1 in 2, antibiotics for 1 in 5 episodes
17667308	19	Netherlands	New residential area, suburb of large city, birth cohort	from 2001 onwards	1 yr	0-1 yrs	687	Diary parents, linked to general practitioner's electronic patient file (childdays of observation not explicitly described)	Average 64.4 ± 60.9SD days with resp symptoms/yr, 47% ≥1 doctor visit for resp symptoms; influenced by child as well as parent characteristics
21591837	20	Netherlands	Retrospective analysis in Netherlands Information Network of General Practice	2002-2008	6 yrs	0-17 yrs	50,000	Incidence rates based on diagnosis codes reported by GPs	0.2% sinusitis, 1.5% AOM, 2.5% serous otitis, tonsillitis, tonsillar hypertrophy incidence age 0-4 yrs; antibiotics in 60% of sinusitis and tonsillitis, 50% AOM, tonsillar hypertrophy 20%, serous otitis 9%; in 90% amoxicillin; referral to ENT surgeon in 1% for sinusitis and 30% for tonsillar hypertrophy, mainly age 0-4 yrs

30804033	21	Australia	Retrospective analysis of ED records in Western Australia	2002-2012	10 yrs	0-16 yrs	1.6m Emergency Department presentations	Emergendy Department data linked to birth cohort data set	26% of ED presentations for RTI (n=418,755), <5 yrs 32% for RTI (n=332 149); 90,421 with repeat RTI presentations (range 1-85x)
18435478	22	Denmark	Large city, maternity ward	2004-2006	1 yr	0-12 ms	228	Inclusion of 20 children per month; structured diary parents, monthly home visit (80,013 childdays of observation)	Resp symptoms 20% of days 0-6 ms, 30% 6-9 ms, 40% 9-12 ms; nasal discharge and cough most prevalent; 83% of children ≥1 doctor visit. risk factors age, season, size of household and residence, day care, family size
25849847	23	Netherlands	New residential area, suburb of large city, birth cohort	from 2008 onwards	12 ms	0-2 up to 12-14 ms	1260	Diary parents, linked to general practitioner's electronic patient file (1,029 childyears of observation)	Focus on otitis media (fever + ≥1 ear pain, ear discharge); 32% ≥1 episode in 1st yr; doctor visit in 82% episodes, antibiotics in 88% of these
30126046	24	Finland	Birth cohort, maternity health care clinics or delivery wards in south-west Finland	2008-2010	2 yrs	0-2 yrs	646	Daily diary, study clinic visits during RTIs and AOM; MacArthur Communicative Development Inventory, 13 & 24 ms (childdays of observation not explicitly described)	9.6% had recurrent respiratory tract infections and 9.9% had recurrent acute otitis media; sociodemographic data were associated with vocabulary development and with the prevalence of recurrent RTIs and AOM
27455443	25	Finland	Birth cohort, maternity health care clinics or delivery wards in south-west Finland	2008-2010	2 yrs	0-2 yrs	1089	Daily diary, study clinic visits during RTIs and AOM; nasal swabs during RTI in 714 children	p90 in number of annual respiratory illness days = 98; >p90 (n=109) median of 9.6 acute RTI per year, 60% of them ≥3 AOM, 70% ≥3 antibiotic courses, 21% hospitalized for acute RTI; older siblings increased risk of recurrent RTI

27228308	26	Australia	Large city, ORChID birth cohort	2010-2014	2 yrs	0-2 yrs	154	Daily diary cards, mailed collectively end of each month, separate burden diary for impact of more severe episodes (88,032 childdays of observation)	2/3 1st born, 1/2 from advantaged families; resp symptoms 20% of day, mean 13 episodes in 1st 2 yrs of life; doctor visit in 28%, antibiotics in 13% of episodes; main symptoms nasal congestion/ discharge and cough, fever and wheeze less common
28137317	27	Sweden	Age-stratified random sampling from the population registry	2013-2014	1 yr	all ages (children and adults)	3245	Weekly questionnaires, follow-up questionnaire in case of symptoms (all online by email) (childweeks of observation not explicitly described)	Focus on symptoms fitting acute gastrointestinal illness, acute respiratory illness and influenza-like illness episodes
30208077	28	Switzerland	Large city, maternity hospitals & obstetrician's practices, BILD birth cohort	2015	1 yr	0-12 ms	487	Interview on history in 1st ms, weekly telephone interview on respiratory symptoms and infections (childweeks of observation not explicitly described)	No association between quintiles of neighborhood child density or interaction with older siblings and number of respiratory symptoms
29712621	29	UK	Registration of Royal College of General Practitioners Research and Surveillance Centre	4 May 2015 - 8 May 2016	1 yr	0-24 yrs	155 general practices	Retrospective review of structured diagnosis codes related to doctor visits in the electronic patient file system	Boys up to 15 yrs presented more with LRTI (mainly acute bronchitis), and boys 5-14 yrs more with allergic rhinitis and asthma than girls

30670390	30	UK	Large city and surrounding 10-mile area, recruitment by GPs in their practice	2016	Up to and including 1st RTI episode	children	485 children in 331 families	Weekly online question (RTI?), if yes, daily questionnaire until symptoms resolved (childweeks of observation not explicitly described)	RTI symptoms lasted up to 3 weeks in 90% of children

[1]PMID = the unique identifier number used in PubMed. [2]Ref = literature reference number in the paper. Extensive PubMed search plus (‘respiratory symptoms’ and ‘children’; snowball method; only studies that did not preselect the cohort for specific risk factors or focused on predictors that are generally not known in the real-life day-to-day situation in the first lines of care were included. AOM = acute otitis media, ED = emergency department, FU = follow-up, GP = general practitioner, (L)RTI(s) = (lower) respiratory tract infection(s), ms = months, n.a. = not applicable, OR = odds ratio, p = percentile, pos = positive, resp = respiratory, wks = weeks, yr(s) = year(s).
