# Supplementary material for: Respiratory Symptoms in Post-infancy Children. A Dutch Pediatric Cohort Study
Source: Front Pediatr. 2020 Dec 17;8:583630. doi: 10.3389/fped.2020.583630 (PMC7773946; doi:10.3389/fped.2020.583630)
Supplement: Supplementary file 2 [file Table_2.pdf]

Supplementary Table 2. Online questionnaire*

**Questionnaire at the start of the study**	
Date of birth	dd/mm/yyyy
Gender	male / female
First 2 digits of postal code	*(Dutch postal codes contain 4 digits followed by 2 letters; the first 2 digits define the geographical area, but give no clue to the exact address)*
Ethnicity	Original Dutch / Surinam / Netherlands Antilles / Indonesian / Moluccan / Moroccan / Turkish / Other Asian / Other African / Latino / Mixed / I prefer not to answer this question
Allergy, asthma or eczema in the family?	no / yes / don't know
Frequent infections in the family?	no / yes / don't know
Smoking in the home?	no / no, only outside / yes, but only by visitors / yes, by family members
**Weekly questionnaire (weeks 1-104)**	
Did your child have any complaints last week?	no / yes
If no: end of questionnaire for that week. If yes: the questions below followed	
Did you visit a doctor (please tick applicable items)?	no / yes, general practitioner / yes, paediatrician / yes, ENT-specialist / yes, other
Did your child get antibiotics?	no / yes
What were the complaints (please tick applicable items)?	earache / ear discharge / throat ache / blocked nose / runny nose / headache / hoarse voice / cough / dyspnoea
Was there a fever ≥ 38.5°C?	no / yes / didn't take the temperature
Did the child miss school?	no / yes / not applicable
Did the child miss study work placement (*'stage' in Dutch*)?	no / yes / not applicable
Did the child miss work?	no / yes / not applicable
Did the parents miss their work?	no / yes / not applicable

*Original questionnaires *in Dutch*. Population cohort is explained in the methods section of the manuscript. ENT = ear-nose-throat.
