# Supplementary material for: Respiratory Symptoms in Post-infancy Children. A Dutch Pediatric Cohort Study
Source: Front Pediatr. 2020 Dec 17;8:583630. doi: 10.3389/fped.2020.583630 (PMC7773946; doi:10.3389/fped.2020.583630)
Supplement: Supplementary file 3 [file Table_3.pdf]

Supplementary Table 3. Duration of episodes of consecutive childweeks with symptoms in the cohort, counted per individual child.

total consecutive episodes with *any symptom* lasting:	in all children together	children <5 years (n=247)	children 5-10 years (n=281)	children ≥10 years (n=227)	total consecutive episodes with *cough* lasting:	in all children together	children <5 years (n=247)	children 5-10 years (n=281)	children ≥10 years (n=227)
1 week	3770	1100	1700	970	1 week	1468	712	424	332
2 weeks	1057	669	112	276	2 weeks	602	344	158	100
3 weeks	291	167	70	54	3 weeks	120	69	30	21
4 weeks	131	63	42	26	4 weeks	56	31	13	12
5 weeks	62	35	17	10	5 weeks	32	23	9	0
6 weeks	39	23	11	5	6 weeks	12	9	2	1
7 weeks	23	15	7	1	7 weeks	8	5	3	0
8 weeks	12	6	4	2	8 weeks	3	2	0	1
9 weeks	7	4	2	1	9 weeks	1	1	0	0
10 weeks	7	5	2	0	10 weeks	2	1	1	0
11 weeks	4	2	1	1	11 weeks	0	0	0	0
12 weeks	2	2	0	0	12 weeks	0	0	0	0
13 weeks	0	0	0	0	13 weeks	0	0	0	0
14 weeks	2	2	0	0	14 weeks	0	0	0	0
15 weeks	0	0	0	0	15 weeks	0	0	0	0
16 weeks	2	2	0	0	16 weeks	1	1	0	0
17 weeks	0	0	0	0	17 weeks	0	0	0	0
20 weeks	1	1	0	0	20 weeks	0	0	0	0
21 weeks	0	0	0	0	21 weeks	0	0	0	0
22 weeks	0	0	0	0	22 weeks	0	0	0	0
62 weeks	1	0	1	0	62 weeks	0	0	0	0
total consecutive episodes with *runny nose* lasting:	in all children together	children <5 years (n=247)	children 5-10 years (n=281)	children ≥10 years (n=227)	total consecutive episodes with *headache* lasting:	in all children together	children <5 years (n=247)	children 5-10 years (n=281)	children ≥10 years (n=227)

1 week	986	528	221	237	1 week	1260	246	455	511
2 weeks	357	233	77	47	2 weeks	154	49	56	73
3 weeks	68	48	13	7	3 weeks	25	10	3	12
4 weeks	29	21	5	3	4 weeks	7	2	4	1
5 weeks	13	11	1	1	5 weeks	2	0	2	0
6 weeks	9	7	2	0	6 weeks	1	0	1	0
7 weeks	2	1	1	0	7 weeks	4	1	3	0
8 weeks	3	3	0	0	8 weeks	0	0	0	0
9 weeks	2	2	0	0	9 weeks	2	0	1	1
10 weeks	1	1	0	0	10 weeks	0	0	0	0
11 weeks	0	0	0	0	11 weeks	0	0	0	0
12 weeks	0	0	0	0	12 weeks	1	0	1	0
13 weeks	0	0	0	0	13 weeks	0	0	0	0
14 weeks	0	0	0	0	14 weeks	0	0	0	0
15 weeks	0	0	0	0	15 weeks	0	0	0	0
16 weeks	0	0	0	0	16 weeks	1	0	1	0
17 weeks	0	0	0	0	17 weeks	0	0	0	0
20 weeks	1	1	0	0	20 weeks	0	0	0	0
21 weeks	0	0	0	0	21 weeks	0	0	0	0
22 weeks	0	0	0	0	22 weeks	1	0	1	0
62 weeks	0	0	0	0	62 weeks	0	0	0	0
total consecutive episodes with *blocked nose* lasting:	in all children together	children <5 years (n=247)	children 5-10 years (n=281)	children ≥10 years (n=227)	total consecutive episodes with *throat ache* lasting:	in all children together	children <5 years (n=247)	children 5-10 years (n=281)	children ≥10 years (n=227)
1 week	1345	593	349	403	1 week	1209	395	394	420
2 weeks	452	221	144	87	2 weeks	251	104	66	81
3 weeks	81	34	37	10	3 weeks	39	17	12	10
4 weeks	24	15	8	1	4 weeks	11	1	7	3
5 weeks	11	6	5	0	5 weeks	6	4	0	2
6 weeks	10	4	6	0	6 weeks	2	1	0	1
7 weeks	4	2	2	0	7 weeks	0	0	0	0

8 weeks	2	2	0	0	8 weeks	0	0	0	0
9 weeks	2	2	0	0	9 weeks	0	0	0	0
10 weeks	0	0	0	0	10 weeks	0	0	0	0
11 weeks	1	1	0	0	11 weeks	0	0	0	0
12 weeks	0	0	0	0	12 weeks	0	0	0	0
13 weeks	0	0	0	0	13 weeks	0	0	0	0
14 weeks	0	0	0	0	14 weeks	0	0	0	0
15 weeks	0	0	0	0	15 weeks	0	0	0	0
16 weeks	0	0	0	0	16 weeks	0	0	0	0
17 weeks	0	0	0	0	17 weeks	0	0	0	0
20 weeks	0	0	0	0	20 weeks	0	0	0	0
21 weeks	0	0	0	0	21 weeks	0	0	0	0
22 weeks	0	0	0	0	22 weeks	0	0	0	0
62 weeks	0	0	0	0	62 weeks	0	0	0	0
total consecutive episodes with *earache* lasting:	in all children together	children <5 years (n=247)	children 5-10 years (n=281)	children ≥10 years (n=227)	total consecutive episodes with *ear discharge* lasting:	in all children together	children <5 years (n=247)	children 5-10 years (n=281)	children ≥10 years (n=227)
1 week	422	229	128	65	1 week	90	52	26	12
2 weeks	101	58	29	14	2 weeks	30	16	13	1
3 weeks	17	8	7	2	3 weeks	7	4	3	0
4 weeks	9	4	5	0	4 weeks	3	1	2	0
5 weeks	3	1	1	1	5 weeks	1	1	0	0
6 weeks	1	1	0	0	6 weeks	0	0	0	0
7 weeks	1	1	0	0	7 weeks	0	0	0	0
8 weeks	0	0	0	0	8 weeks	0	0	0	0
9 weeks	0	0	0	0	9 weeks	0	0	0	0
10 weeks	0	0	0	0	10 weeks	0	0	0	0
11 weeks	0	0	0	0	11 weeks	0	0	0	0
12 weeks	0	0	0	0	12 weeks	0	0	0	0
13 weeks	0	0	0	0	13 weeks	0	0	0	0
14 weeks	0	0	0	0	14 weeks	0	0	0	0

15 weeks	0	0	0	0	15 weeks	0	0	0	0
16 weeks	0	0	0	0	16 weeks	0	0	0	0
17 weeks	0	0	0	0	17 weeks	0	0	0	0
20 weeks	0	0	0	0	20 weeks	0	0	0	0
21 weeks	0	0	0	0	21 weeks	0	0	0	0
22 weeks	0	0	0	0	22 weeks	0	0	0	0
62 weeks	0	0	0	0	62 weeks	0	0	0	0
total consecutive episodes with *dyspnea* lasting:	in all children together	children <5 years (n=247)	children 5-10 years (n=281)	children ≥10 years (n=227)	total consecutive episodes with *hoarse voice* lasting:	in all children together	children <5 years (n=247)	children 5-10 years (n=281)	children ≥10 years (n=227)
1 week	273	122	82	63	1 week	497	213	138	146
2 weeks	38	22	12	8	2 weeks	93	39	23	31
3 weeks	9	3	5	1	3 weeks	15	8	5	2
4 weeks	3	0	3	0	4 weeks	2	1	1	0
5 weeks	1	0	1	0	5 weeks	2	0	0	2
6 weeks	0	0	0	0	6 weeks	0	0	0	0
7 weeks	1	0	1	0	7 weeks	0	0	0	0
8 weeks	0	0	0	0	8 weeks	0	0	0	0
9 weeks	0	0	0	0	9 weeks	0	0	0	0
10 weeks	0	0	0	0	10 weeks	0	0	0	0
11 weeks	0	0	0	0	11 weeks	0	0	0	0
12 weeks	0	0	0	0	12 weeks	0	0	0	0
13 weeks	0	0	0	0	13 weeks	0	0	0	0
14 weeks	0	0	0	0	14 weeks	0	0	0	0
15 weeks	0	0	0	0	15 weeks	0	0	0	0
16 weeks	0	0	0	0	16 weeks	0	0	0	0
17 weeks	0	0	0	0	17 weeks	0	0	0	0
20 weeks	0	0	0	0	20 weeks	0	0	0	0
21 weeks	0	0	2	0	21 weeks	0	0	0	0
22 weeks	2	0	0	0	22 weeks	0	0	0	0
62 weeks	0	0	0	0	62 weeks	0	0	0	0

total consecutive episodes with *fever* lasting:	in all children together	children <5 years (n=247)	children 5-10 years (n=281)	children ≥10 years (n=227)					
1 week	812	489	241	82					
2 weeks	142	94	38	10					
3 weeks	13	7	5	1					
4 weeks	1	1	0	0					
5 weeks	2	1	1	0					
6 weeks	0	0	0	0					
7 weeks	0	0	0	0					
8 weeks	0	0	0	0					
9 weeks	0	0	0	0					
10 weeks	0	0	0	0					
11 weeks	0	0	0	0					
12 weeks	0	0	0	0					
13 weeks	0	0	0	0					
14 weeks	0	0	0	0					
15 weeks	0	0	0	0					
16 weeks	0	0	0	0					
17 weeks	0	0	0	0					
20 weeks	0	0	0	0					
21 weeks	0	0	0	0					
22 weeks	0	0	0	0					
62 weeks	0	0	0	0					

This table shows aggregated data (all childweeks of all children). Total reported childweeks n = 55,524 of which 8,425 with complaint(s).In this table, episodes preceded and/or followed by a missing value (week not reported; n = 6,194) were included in the count as if the missing value was ‘no symptoms in that week’. So, in reality the curves of episode duration are shifted slightly towards longer episodes, leading to some underestimation of the duration of symptom blocks. However, this is only a small bias. In comparison, the children with full participation (104 reported childweeks) reported a total of 570, 191, 58, 30, 14, 14, and 13 consecutive episodes of 1, 2, 3, 4, 5, 6, and 7 or more consecutive childweeks with *any symptoms*, respectively, with a weak contingency coefficient (0.053; chi-square, one-sided, p=.012).
