# Supplementary material for: Respiratory Symptoms in Post-infancy Children. A Dutch Pediatric Cohort Study
Source: Front Pediatr. 2020 Dec 17;8:583630. doi: 10.3389/fped.2020.583630 (PMC7773946; doi:10.3389/fped.2020.583630)
Supplement: Supplementary file 4 [file Table_4.pdf]

Supplementary Table 4: A. Latent profile (LPA) and B. principle component (PCA) analysis of the proportion of symptoms in the individual children.

A	***Best fitting number of clusters in LPA*** *(BIC) (number of children that could be included in the analysis)*														
	all children#			*<5 yrs#*			*≥5 yrs#*			*5-10 yrs#*			*≥10 yrs#*		
year-round	9	(31270.22)	(712)	9	(8840.674)	(228)	8	(21050.3)	(484)	9	(11171.13)	(267)	8	(9533.248)	(217)
spring	7	(23392.35)	(569)	2	(7433.35)	(196)	8	(17078.08)	(373)	4	(6854.166)	(209)	4	(6715.52)	(164)
summer	9	(13427.94)	(426)	9	(13427.94)	(152)	2	(10677.43)	(274)	8	(4948.667)	(144)	2	(5696.744)	(130)
autumn	5	(13710.9)	(400)	3	(4863.445)	(148)	4	(9062.606)	(252)	2	(4473.168)	(142)	3	(3663.155)	(110)
winter	9	(17364.88)	(559)	9	(7049.492)	(177)	9	(12784.5)	(382)	4	(7014.73)	(209)	7	(4902.725)	(173)
seasons combined	9	(32817.67)	(400)	6	(14346.51)	(196)	9	(17129.72)	(204)	9	(17129.72)	(204)	-	-	(0)

B	***Components in PCA**** *(loadings)*									
year-round	*all children (n=712)*		*<5 yrs (n=228)*		*≥5 yrs (n=484)*		*5-10 yrs (n=267)*		*≥10 yrs (n=217)*	
	co, rn, bn	(0.790, 0.782, 0.798)	co, rn, bn	(0.831, 0.853, 0.800)	co, rn, bn	(0.804, 0.743, 0.769)	co, rn, bn	(0.804, 0.774, 0.663)	co, rn, bn	(0.802, 0.697, 0.814)
	ta, ha, ed	(0.646, 0.689, 0.675)							hv, dy	(0.833, 0.807)
spring	*all children (n=569)*		*<5 yrs (n=196)*		*≥5 yrs (n=373)*		*5-10 yrs (n=209)*		*≥10 yrs (n=164)*	
	co, rn, bn	(0.798, 0.798, 0.798)	co, rn, bn	(0.850, 0.778, 0.791)	co, rn, bn	(0.775, 0.789, 0.755)	co, bn, ta, ea	(0.626, 0.805, 0.624, 0.617)	co, rn, bn	(0.792, 0.883, 0.760)
summer	*all children (n=426)*		*<5 yrs (n=152)*		*≥5 yrs (n=274)*		*5-10 yrs# (n=287)*		*≥10 yrs (n=130)*	
	co, rn, bn	(0.811, 0.836, 0.761)	ta, ha, ea, hv, ed	(0.680, 0.665, 0.625, 0.673, 0.802)	co, rn, bn	(0.832, 0.883, 0.799)	*co, rn, bn*	*(0.783, 0.790, 0.783)*	co, rn, bn	(0.868, 0.906, 0.818)
	ha, ea, ed	(0.632, 0.673, 0.662)	co, rn	(0.759, 0.777)						
autumn	*all children (n=400)*		*<5 yrs (n=148)*		*≥5 yrs (n=252)*		*5-10 yrs (n=142)*		*≥10 yrs (n=110)*	
	co, rn, bn	(0.788, 0.7777, 0.795)	co, rn, bn, ea	(0.783, 0.761, 0.800, 0636)	co, rn, bn	(0.820, 0.797, 0.747)	co, rn, bn, ea	(0.823, 0.745, 0.843, 0.608)	co, rn, hv	(0.834, 0.631, 0.720)
							ta, ha	(0.715, 0.835)		
winter	*all children (n=559)*		*<5 yrs (n=177)*		*≥5 yrs (n=382)*		*5-10 yrs (n=209)*		*≥10 yrs (n=173)*	
	co, rn, bn, ea	(0.724, 0.815, 0.816, 0.677)	co, rn, bn	(0.742, 0.789, 0.813)	co, rn, bn, ea	(0.788, 0.807, 0.809, 0.667)	co, rn, bn, ta, ea	(0.710, 0.800, 0.847, 0.691, 0.717)	co, rn, bn	(0.601, 0.837, 0.665)
							hv, dy, ea	(0.824, 0.711, 0.800)		

C	*seasons combined*									
	all children (n=239)		*<5 yrs (n=97)*		*≥5 yrs (n=142)*		*5-10 yrs$^{\#}$ (n=287)*		*≥10 yrs$^{\#}$ (n=228)*	
Components in PCA* *(loadings)*	co-sp, co-au, rn-sp, rn-su, rn-au, bn-au	(0.615, 0.636, 0.751, 0.751, 0.811, 0.645)	co-au, bn-sp, bn-su, bn-au	(0.679, 0.698, 0.628, 0.825)	rn-sp, rn-su, rn-au, bn-au	(0.806, 0.659, 0.843, 0.671)	*co-au, rn-sp, rn-su, rn-au, bn-au*	*(0.721, 0.761, 0.810, 0.881, 0.639)*	*ha-sp, ha-su, ha-au, ha-wi*	*(0.832. 0.865, 0.805, 0.677)*
	ha-sp, ha-su, ha-au, ha-wi	(0.784, 0.716, 0.790, 0.610,)	rn-sp, rn-su, rn-au, rn-wi	(0.698, 0.675, 0.689, 0.809)	ha-sp, ha-su, ha-au, ha-wi	(0.753, 0.815, 0.845, 0.667)	*bn-wi, ta-wi, ea-wi, hv-au,*	*(0.665, 0.741, 0.787, 0.796)*	*co-sp, rn-sp, rn-au, bn-sp, bn-au,*	*(0.617, 0.812, 0.723, 0.650, 0.674)*
			dy-su, dy-au, dy-wi	(0.770, 0.934, 0.839)	co-wi, bn-wi, ta-wi	(0.769, 0.737, 0.741)	*ta-sp, ta-su*	*(0.676, 0.741)*	*co-su, rn-su, bn-su*	*(0.862, 0.843, 0.806)*

Latent profile analysis (LPA) and principal component analysis (PCA) were performed separately on the proportion (per all reported childweeks, per individual child) of ‘yes, this symptom is present’ determined either year-round, or per separate season (LPA: A, PCA: B), or on the proportion (per all reported childweeks, per individual child) of ‘yes, this symptom is present’ determined per season, grouped together in one combined analysis (LPA: A, PCA: C). Only components with loadings ≥0.600 were included in the PCA. Missing values deleted listwise in the LPA and pairwise in the PCA except when # is shown, then missing values were replaced with the mean in the PCA in this subgroup because of too few data remaining for PCA otherwise. *Only components obtained in PCA analysis with Eigenvalue located above the point where the scree plot of the Eigenvalues tapered off are shown. For each subgroup the number of children that could be included in the PCA is shown. Abbreviations: au, autumn; BIC, Bayesian information criterion; bn, blocked nose; co, cough; dy, dyspnea; ea, earache; ed, ear discharge; fe, fever; ha, headache; hv, hoarse voice; rn, runny nose; sp, spring; su, summer; ta, throat ache; wi, winter; yrs, years.

A. The best fitting number of clusters in the LPA ranged from 2 to 9. **B.** The components in the PCA (depicted in the rows) comprise combinations of proportions of childweeks with ‘yes, this type of symptom is present’ for ‘year-round’ as well as separate ‘per season’ analysis. **C.** The components in the PCA (depicted in the rows) comprise combinations of proportions of childweeks with ‘yes, this type of symptom is present in this season’ for ‘season-combined’, the combined analysis of the proportions determined per type of symptom per season (e.g. co-sp = the proportion of ‘yes, cough is present’ in the childweeks reported in spring).
