# Supplementary material for: Respiratory Symptoms in Post-infancy Children. A Dutch Pediatric Cohort Study
Source: Front Pediatr. 2020 Dec 17;8:583630. doi: 10.3389/fped.2020.583630 (PMC7773946; doi:10.3389/fped.2020.583630)
Supplement: Supplementary file 6 [file Data_Sheet_1.pdf]

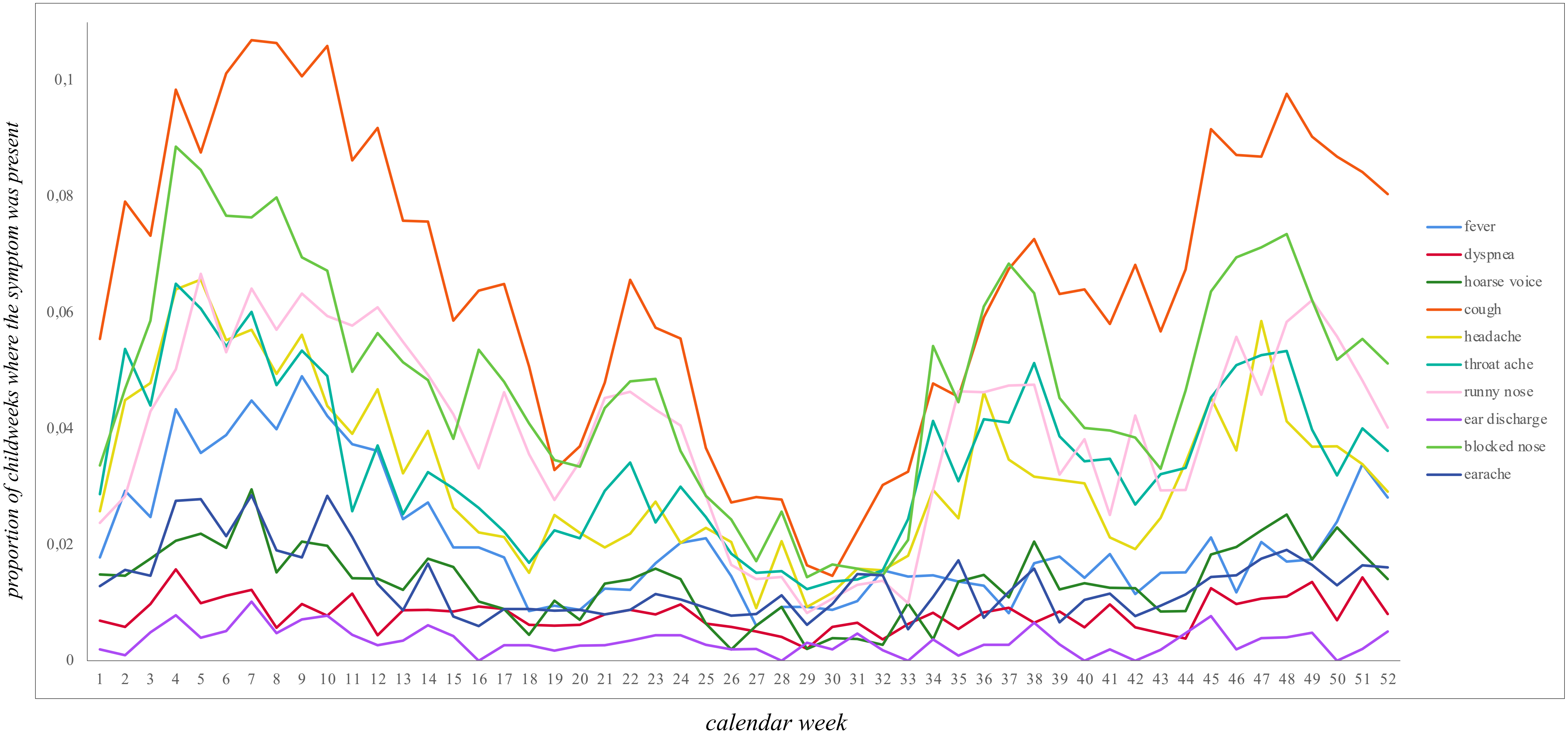

Supplementary Figure 1A. Proportion of childweeks where the symptom was 'yes, present' depicted per calendar week (1 to 52; aggregated information from years 2012 to 2015; all children and all childweeks taken together).

All symptoms could be reported as 'yes, present' or 'no, not present', except fever which could be reported as 'temperature not taken' as well. Although the various symptoms show different seasonal patterns, note the very low overall proportions of 'yes, present' for all symptoms, even in winter: the y-axis is only depicted up to 0.1.

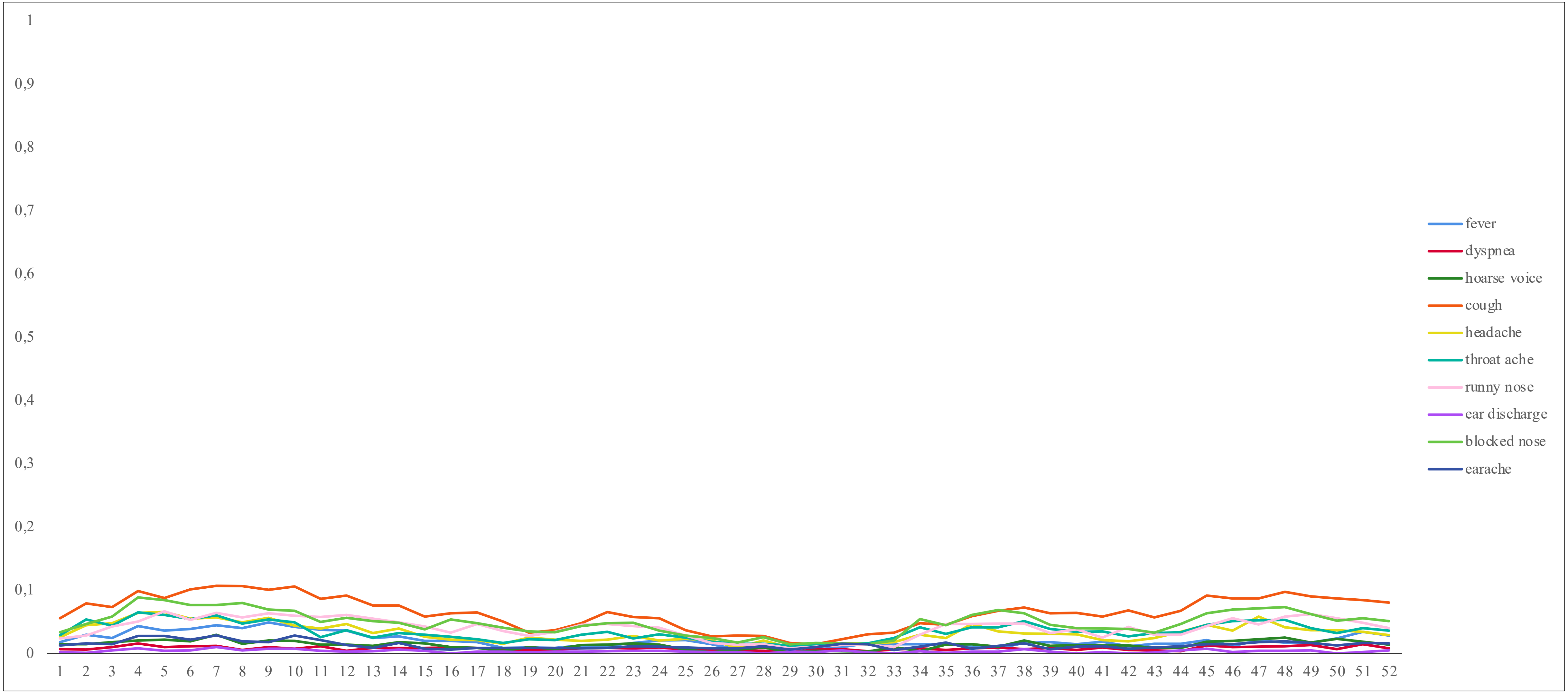

Supplementary Figure 1B. The same as figure 2A, but now, the y-axis is depicted in the full range of proportions from 0 to 1. This clearly illustrates the limited influence of season on the various symptoms in this cohort of children from the general population.

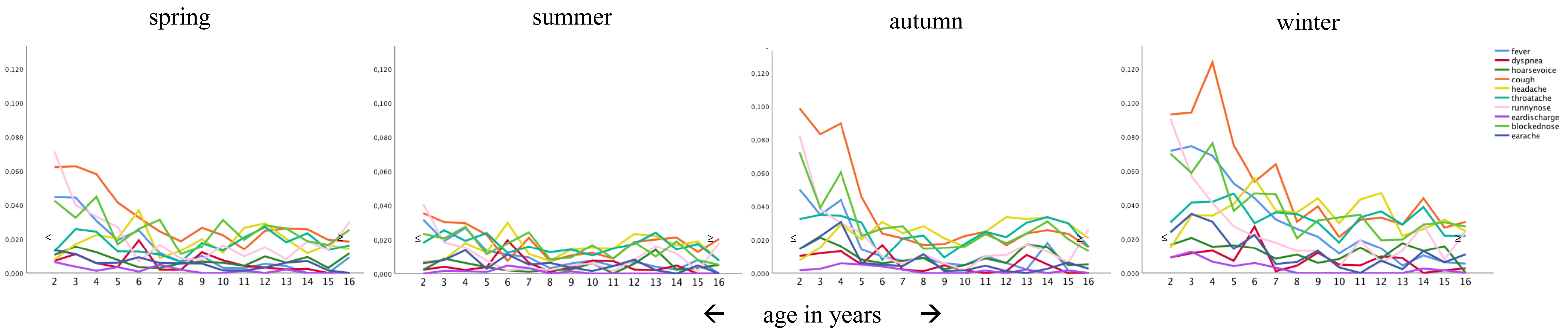

Supplementary Figure 1C. Proportion of childweeks where the symptom was 'yes, present' (y-axis) related to age at inclusion (x-axis), depicted per season (aggregated information from years 2012 to 2015; all children and all childweeks taken together).

All symptoms could be reported as 'yes, present' or 'no, not present', except fever which could be reported as 'temperature not taken' as well. Note the seasonal pattern is most pronounced in the younger children, albeit that the seasonal influence is small (note the depicted range of the y-axis until 0.120, and see Supplementary Figure 3B).
